# Social disparities and the COVID-19, influenza, and RSV ‘tripledemic’ in Ontario, Canada: A population-based study

**DOI:** 10.1017/S0950268826102131

**Published:** 2026-09-24

**Authors:** Evan Mitchell, Haiyan Zhu, Tiffany Fitzpatrick, Aditi Majoe, Naheemot Olaoluwa Sule, Roberto Guglielmi, Zahid Ahmad Butt

**Affiliations:** 1 Applied Mathematics, University of Waterloo, Canada; 2 University of Toronto Dalla Lana School of Public Health, Canada; 3 School of Public Health Sciences, University of Waterloo, Canada; 4 Institute for Clinical Evaluative Sciences, Canada

**Keywords:** age-specific hospitalization rates, neighbourhood-level ethno-racial diversity, neighbourhood-level material deprivation, rurality, sex

## Abstract

We examined inequalities in weekly hospitalization rates related to COVID-19, influenza, and respiratory syncytial virus based on neighbourhood-level material deprivation, neighbourhood-level ethno-racial diversity, rurality, and sex. Hospitalization rates were measured as the weekly number of hospitalizations per 10 000 people. Local regression models were used to fit observed weekly hospitalization rates within each study year and each population subgroup, and statistical significance of between-subgroup differences in peak hospitalization rate was assessed based on the overlap of 95% confidence intervals. We also provided comparisons of pre-pandemic (July 2017–June 2020) versus post-pandemic (July 2020–June 2023) trends. More materially deprived neighbourhoods showed up to a 2.5-times increase in peak weekly hospitalization rates compared to less materially deprived neighbourhoods. Peak hospitalization rates were up to 2.3-times higher among more versus less diverse neighbourhoods. Some age groups showed peak hospitalization rates up to 2.8-times higher among urban residents. Peak hospitalization rates were up to 1.3-times higher in younger males versus younger females and up to 1.6-times higher in older females versus older males. Variations due to neighbourhood-level material deprivation, neighbourhood-level ethno-racial diversity, rurality, and sex should be considered when designing and implementing public health policies aiming to reduce respiratory virus burden.

## Introduction

Respiratory syncytial virus (RSV), influenza, and COVID-19 are all airborne viral pathogens with similar transmission mechanisms and some overlapping symptoms. Influenza and RSV place substantial strain upon the Canadian healthcare system annually [1–5], which was further compounded by the COVID-19 pandemic [6, 7]. In 2021, relaxation of pandemic-related non-pharmaceutical interventions (NPIs) and an atypical respiratory virus season led to fears globally of healthcare infrastructures being overwhelmed by the ‘tripledemic’ of the combined burden of RSV, influenza, and COVID-19 infections [8, 9].

While RSV presents with mild symptoms for most people, infants and people over the age of 65 are at increased risk of developing severe cases and are more likely to require hospitalization [3]. Influenza and COVID-19, by contrast, tend to be less severe in younger children but show increased severity in those aged 65 years and older [2]. In addition to age, studies suggest sex to be a biological risk factor for severe disease across many pathogens [10] with strong evidence specifically for influenza [11], RSV [12], and COVID-19 [13, 14]. Social factors such as ethno-racial identity and socioeconomic status have also been shown to play a role in respiratory disease outcomes [15–19]. Some studies on the effects of material deprivation on respiratory illness-related hospitalization have suggested a positive relationship between the two [20]. Studies have also shown that infection rates for many respiratory viruses are higher among more population-dense areas, due to an increased number and frequency of contacts within the population [21, 22].

Most of the studies mentioned above focused on either a single disease or age group. In this paper, we describe trends in age-specific hospitalization rates due to COVID-19, influenza, or RSV across all age groups using population-based data from Ontario, Canada, with particular emphasis on how these trends changed following the COVID-19 pandemic. Hospitalization rates were further stratified according to the following variables: sex, rurality, neighbourhood-level material deprivation, and neighbourhood-level ethno-racial diversity. Our objective was to elucidate inequalities between these various subgroups of individuals and provide a description of how those inequalities manifest across all age groups, comparing seasons before the COVID-19 pandemic to those after the pandemic.

## Methods

This retrospective cohort study was an analysis of population-based hospital administrative and census data capturing the population of Ontario. Datasets were linked using unique encoded identifiers at ICES, and weekly age-specific hospital admission rates related to influenza, RSV, and COVID-19 were calculated from the last week of June 2017 through the last week of June 2023. Validated algorithms were used to identify these based on ICD10 (International Classification of Diseases) diagnosis codes [23–25]. Rates were calculated as the number of hospitalizations per week per 10 000 individuals and were categorized into six age groups: 0–2, 3–5, 6–12, 13–18, 19–64, and 65+ years old.

To understand the hospitalization patterns within various population subgroups, age-specific rates were further stratified according to: sex, rurality (determined by individual postal code regions), neighbourhood-level material deprivation (measured by access to material resources), and neighbourhood-level ethno-racial diversity (measured by the percentage of the community who are recent newcomers or who self-identify as a visible minority). The last two of these dimensions were measured at the dissemination area level using the Ontario Marginalization Index [26].

For each age group and demographic dimension within each disease, we analysed the data by annual season, defined as July 1–June 30, to capture the annual seasonality of influenza and RSV. Local regression, using a locally estimated scatterplot smoothing method, was applied to each study year, and a 95% confidence interval was constructed around each regression curve. The statistical significance of observed differences between subgroups was determined by assessing overlap of confidence intervals around the seasonal peaks. We also compared hospitalization patterns from before (July 2017–June 2020) and after (July 2020–June 2023) the onset of the COVID-19 pandemic. We compared both the peak weekly hospitalization rate within each season and the average weekly hospitalization rate across each season. Analysis was performed using R (version 4.4.1) and SAS (version 8.3.7.202).

The use of data in this project was authorized under section 45 of Ontario’s Personal Health Information Protection Act and is exempt from research ethics board review.

## Results

### Overall trends and seasonality

**Figure 1.**
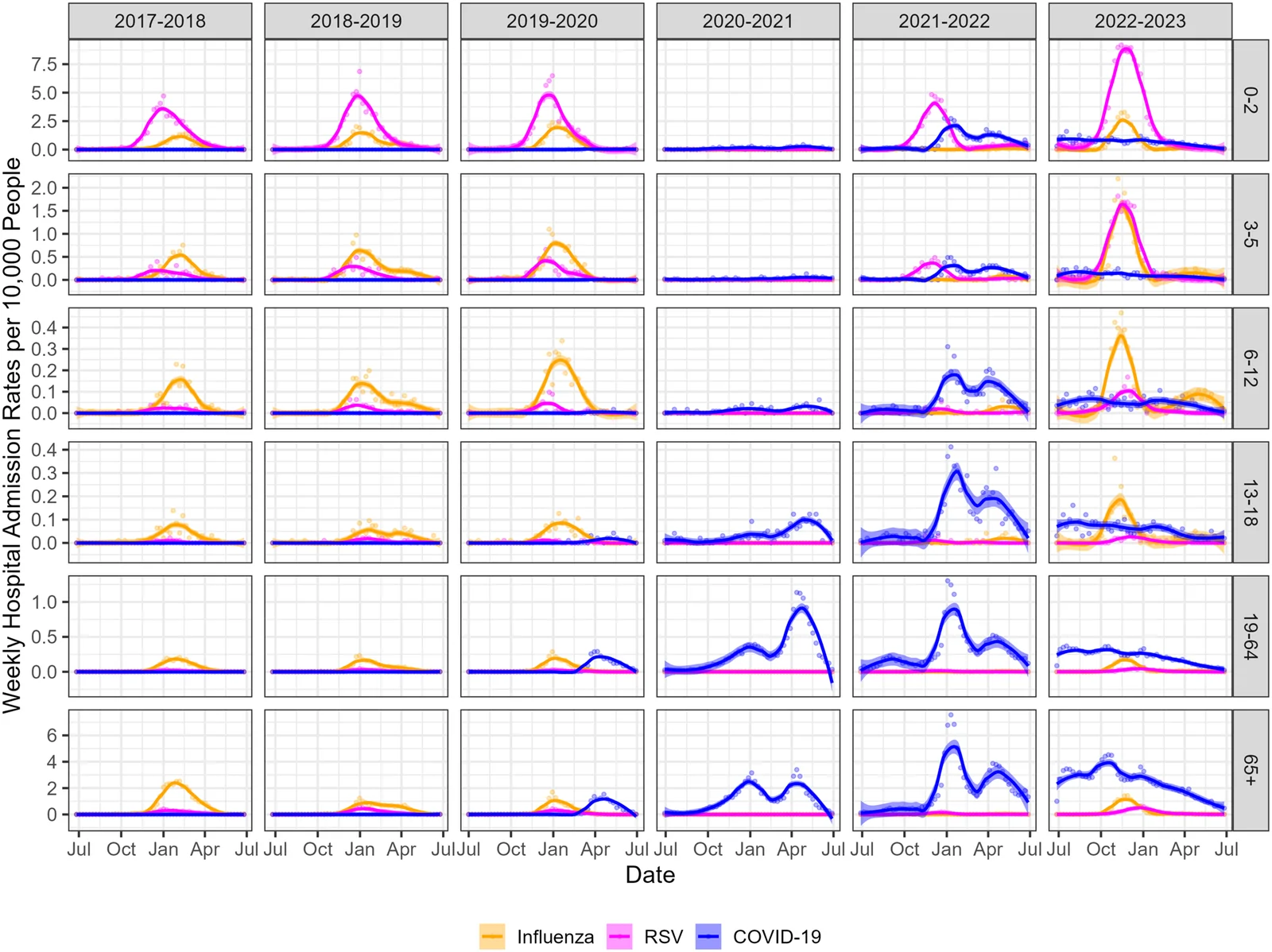
Weekly age-specific hospital admission rates due to influenza (orange), RSV (magenta), and COVID-19 (blue). Points represent observed data, while lines and shaded bands represent local regression curves and associated 95% confidence intervals. *Note:* Vertical axis scales differ between rows. Figure 1. long description.

Figure 1 shows hospitalization rates related to influenza, RSV, and COVID-19 across all age groups and all study years. Influenza and COVID-19 hospitalizations were comparable across all age groups, while RSV hospitalizations predominantly occurred in young children and seniors. In the study years prior to the appearance of COVID-19, influenza hospitalizations started in early November each year and lasted until mid-April of the following year. Hospitalizations from RSV followed a similar pattern, beginning in mid-October each year and lasting until early April of the following year.

During the initial phase of the COVID-19 pandemic in 2020, following the introduction of widespread NPIs in March 2020, hospitalization rates for both influenza and RSV declined to historically low levels across all age groups. In contrast, COVID-19 accounted for a substantial share of respiratory hospitalizations beginning in 2020, particularly among individuals 19–64 and 65+ years old.

In the 2021–2022 season, RSV hospitalization rates largely returned to pre-pandemic levels, although rates among elderly individuals aged 65+ remained below the pre-pandemic baseline. Influenza hospitalizations, however, remained substantially suppressed, reaching only approximately 10% of typical pre-pandemic levels across all age groups.

In the 2022–2023 season, hospitalizations for both influenza and RSV returned to or exceeded pre-pandemic levels. Seasonal timing and duration also shifted. For example, RSV hospitalizations among children 0–5 years old began earlier in late September 2022 and ended in February 2023, peaking at a weekly rate roughly double that observed before the pandemic. Influenza hospitalizations followed a similar trend beginning in late September 2022 and ending in February 2023; children 3–12 years old experienced the largest increase, almost doubling the peak hospitalization rates observed pre-pandemic. COVID-19 hospitalizations remained elevated during this period, contributing to a heightened overall disease burden.

### Neighbourhood-level material deprivation

**Figure 2.**
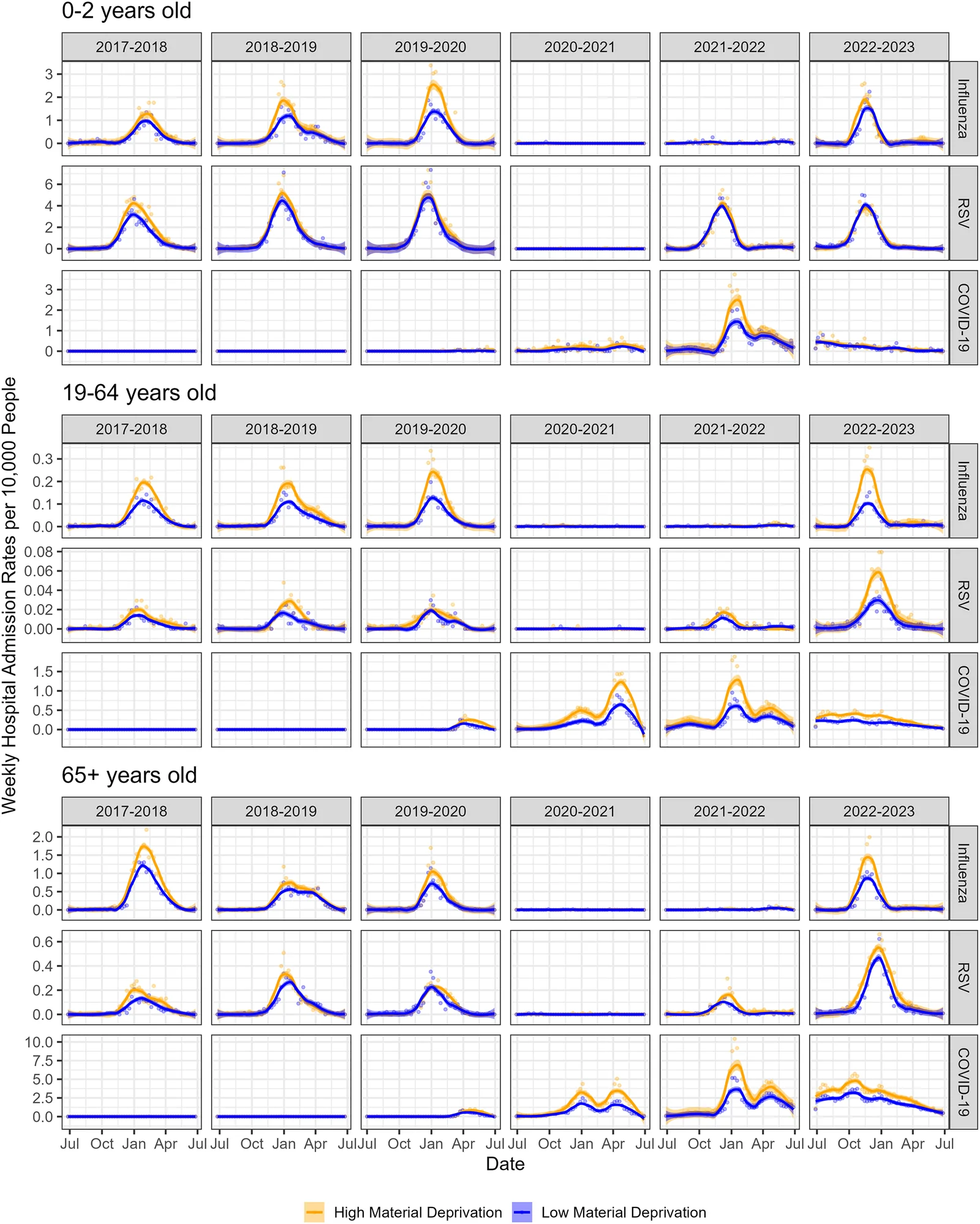
Weekly age-specific hospital admission rates for high (orange) and low (blue) levels of neighbourhood-level material deprivation. Points represent observed data, while lines and shaded bands represent local regression curves and associated 95% confidence intervals. *Note:* Vertical axis scales differ between rows. Figure 2. long description.

Before the COVID-19 pandemic, individuals from highly materially deprived communities experienced significantly higher hospitalization rates from influenza and RSV relative to their less materially deprived counterparts (Figure 2). For influenza, this disparity was most pronounced among individuals 0–2 and 19–64 years old during the 2019–2020 season. Individuals 0–2 and 19–64 years old living in areas with high levels of material deprivation were hospitalized at a peak weekly rate roughly 1.9-times higher and at an average weekly rate roughly 1.7-times higher than that of individuals in less materially deprived neighbourhoods. During the 2018–2019 RSV season, individuals 19–64 years old from highly materially deprived areas experienced a peak weekly hospitalization rate roughly 1.7-times higher and an average weekly hospitalization rate roughly 2.0-times higher than those from less materially deprived areas. Similar disparities were observed during the 2017–2018 RSV season, where individuals 0–2 years old from areas with high levels of material deprivation had a peak hospitalization rate 30% greater and an average weekly hospitalization 27% greater, while individuals 65+ years old from areas with high levels of material deprivation had a peak hospitalization rate 50% greater and an average weekly hospitalization 42% greater than those from less materially deprived areas.

Following the appearance of COVID-19, most of these disparities were still observed. This was most strongly seen among individuals 19–64 years old during the 2022–2023 season of both influenza and RSV. Within this age group, peak and average hospitalization rates due to RSV were roughly doubled in areas with high levels of material deprivation, while peak hospitalization rates due to influenza were roughly 2.5-times higher and average hospitalization rates were roughly 2.3-times higher among those from areas with high levels of material deprivation. Relative peak hospitalization rates in other age groups remained near pre-pandemic levels (Supplementary Figure S1). A similar disparity was seen in COVID-19 hospitalizations in 2021–2022 across all age groups (Supplementary Figure S1); however, by 2022–2023 a statistically significant increase in hospitalizations among those from areas with high levels of material deprivation was only observed among individuals 65+ years old.

### Neighbourhood-level ethno-racial diversity

**Figure 3.**
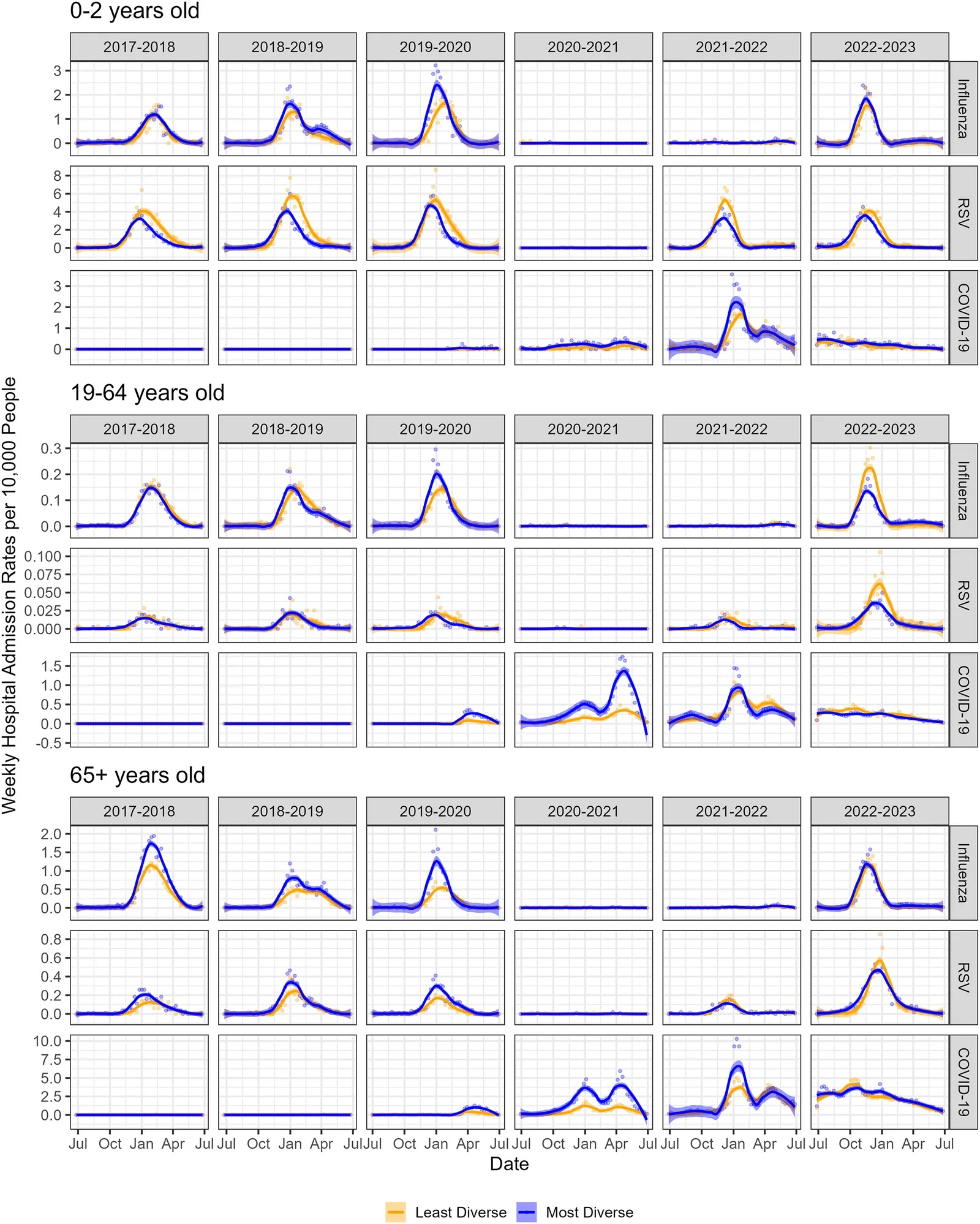
Weekly age-specific hospital admission rates for least (orange) and most (blue) ethno-racially diverse neighbourhoods. Points represent observed data, while lines and shaded bands represent local regression curves and associated 95% confidence intervals. *Note:* Vertical axis scales differ between rows. Figure 3. long description.

Pre-pandemic, statistically significant differences between individuals from more versus less diverse communities varied by age group and study year (Figure 3). Individuals 65+ years old from more ethno-racially diverse neighbourhoods experienced significantly higher rates of hospitalization from both influenza and RSV from 2017 to 2020, with peak weekly rates up to 2.3-times higher for influenza (with seasonal average rates up to 1.8-times higher) and up to 1.8-times higher for RSV (with seasonal average rates up to 1.7-times higher) in more diverse communities relative to less diverse areas. Similar trends existed for influenza hospitalizations in other age groups during specific study years (Supplementary Figure S2). However, RSV hospitalizations showed the reverse pattern in those 0–2 years old from 2017 to 2020, with peak weekly rates up to 1.4-times higher and seasonal average weekly rates up to 1.5-times higher in individuals from less diverse neighbourhoods compared to their more diverse neighbourhood counterparts.

The initial years of the COVID-19 pandemic, namely 2020–2022, showed a similar trend of higher hospitalization rates among more diverse neighbourhoods across all age groups (Supplementary Figure S2). In the 2022–2023 season, though, statistically significant differences between more versus less diverse communities disappeared across all three diseases. The exception being those 19–64 years old, where individuals from less diverse neighbourhoods experienced a peak weekly hospitalization rate roughly 69% higher for influenza (with an average weekly hospitalization rate roughly 42% higher) and 72% higher for RSV (with an average weekly hospitalization rate roughly 40% higher) than individuals from more diverse neighbourhoods.

### Urban versus rural differences

**Figure 4.**
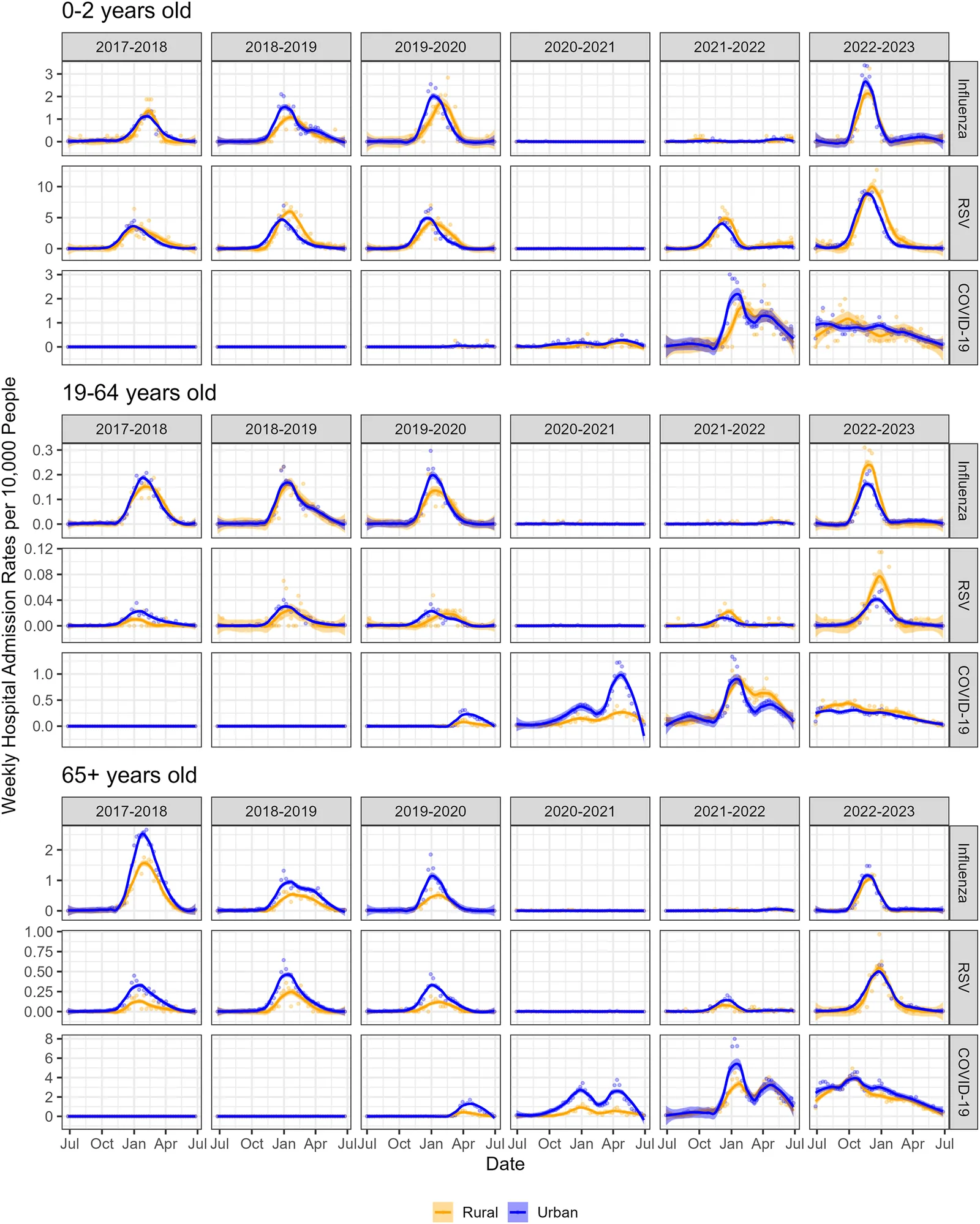
Weekly age-specific hospital admission rates for rural (orange) and urban (blue) residents. Points represent observed data, while lines and shaded bands represent local regression curves and associated 95% confidence intervals. *Note:* Vertical axis scales differ between rows. Figure 4. long description.

Before the pandemic, no statistically significant differences between urban versus rural residents were observed across most age groups (Supplementary Figure S3). The exception was for those 65+ years old, where urban residents showed significantly higher hospitalization rates than rural residents for both influenza and RSV (Figure 4). Peak hospitalization rates were up to 1.6-times higher, and average hospitalization rates were up to 1.4-times higher among urban residents for influenza in the 2017–2018 season, while peak hospitalization rates were up to 2.8-times higher and average hospitalization rates were up to 2.3-times higher among urban residents for RSV in the 2019–2020 season relative to rural residents.

COVID-19 initially showed significantly higher hospitalization rates among urban residents aged 0–2 and 19+ years old. However, by the 2022–2023 season, this trend largely disappeared for all three diseases, and no statistically significant differences between urban and rural residents were observed across most age groups (Supplementary Figure S3). One exception to this was among those 6–12 years old, where hospitalization rates for influenza were significantly higher among urban residents, with a peak rate roughly 1.7-times larger and an average rate roughly 1.8-times larger than among rural residents. A reverse trend appeared in individuals 19–64 years old, where rural residents showed significantly higher hospitalization rates from both influenza and RSV. Peak weekly rates were roughly 1.5-times higher and average weekly rates were roughly 1.7-times higher for influenza, while peak rates were 1.9-times higher and average rates were 1.7-times higher for RSV, than among urban residents.

### Sex differences

**Figure 5.**
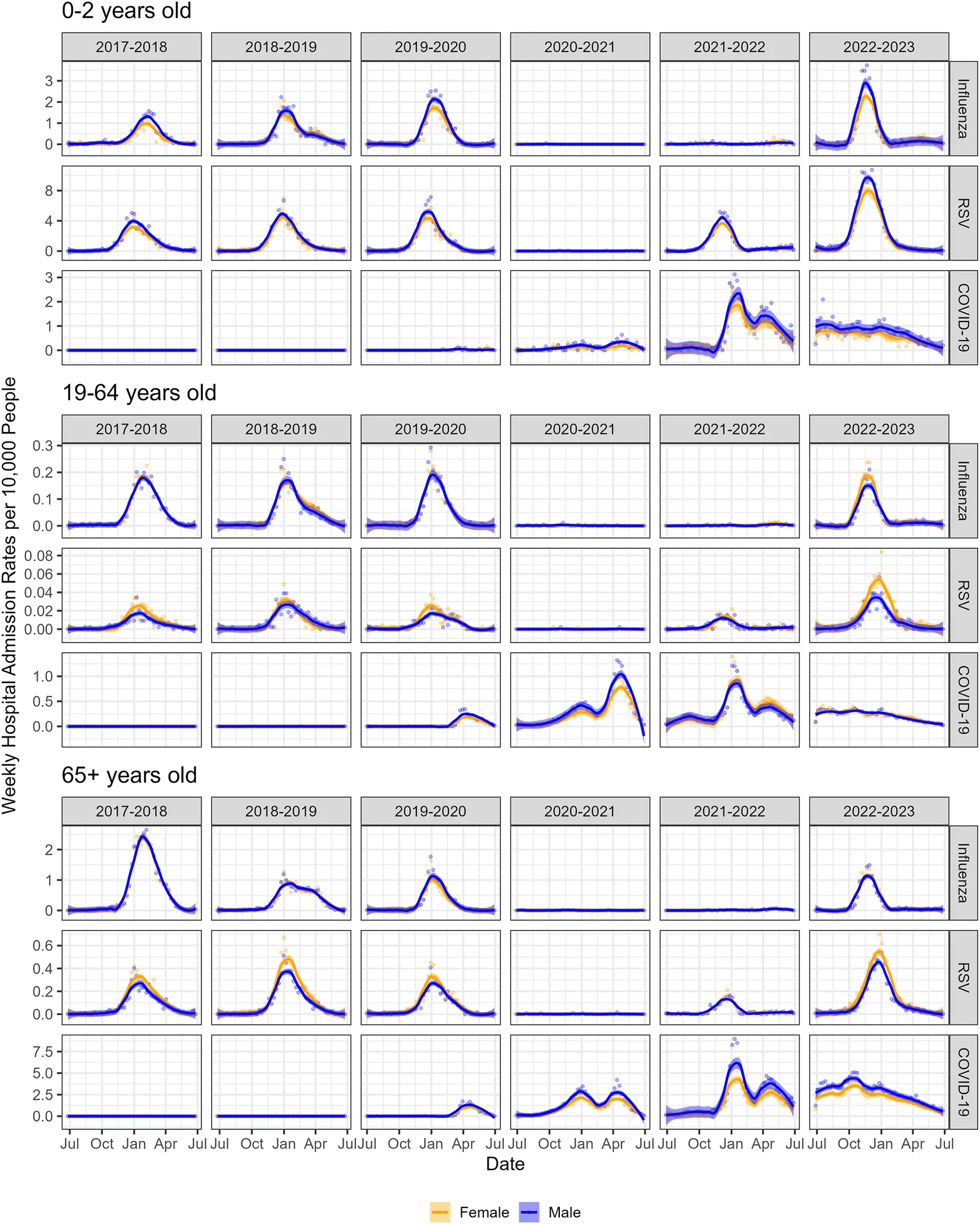
Weekly age-specific hospital admission rates for female (orange) and male (blue) sex. Points represent observed data, while lines and shaded bands represent local regression curves and associated 95% confidence intervals. *Note:* Vertical axis scales differ between rows. Figure 5. long description.

Figure 5 provides evidence of small, but statistically significant, differences between pre-pandemic hospitalization rates among females and males; however, these differences varied by age group. For example, among those 0–2 years old, males experienced slightly higher hospitalization rates from both influenza and RSV, with peak rates up to 20% higher than females. In contrast, in those 65+ years old, there were no significant sex differences for influenza, while females experienced slightly higher hospitalization rates from RSV, with a peak rate roughly 30% higher than in males.

Similar sex-specific trends were also observed for COVID-19, influenza, and RSV following the pandemic (Supplementary Figure S4). Among those 0–2 years old, the peak hospitalization rate during the 2022–2023 season was roughly 1.3-times higher for influenza or 1.2-times higher for RSV in males relative to females, while males also experienced an average weekly hospitalization rate 1.1-times higher for influenza and 1.2-times higher for RSV relative to females. Females 65+ years old experienced a peak hospitalization rate from RSV roughly 1.2-times higher and an average hospitalization rate roughly 1.3-times higher than males in the same age group. Significant differences were also observed in those 19–64 years old, where females had a peak hospitalization rate 1.3-times higher and an average hospitalization rate 1.2-times higher for influenza, and a peak rate and average rate 1.6-times higher for RSV, than males.

## Discussion

In this study, we examined weekly age-specific hospitalization rates due to RSV, influenza, and COVID-19 across multiple seasons, capturing the entire population of Ontario, Canada. We analysed the data across various sociodemographic factors – neighbourhood-level material deprivation, neighbourhood-level diversity, rurality, and sex – to describe statistically significant differences between various population subgroups. These results suggested that age, disease, material deprivation, diversity, rurality, and sex interacted in manifold ways to impact hospitalization rates, and these interplays should be considered when designing and implementing public health policy.

Our findings related to material deprivation and diversity agreed with and expanded on those from previous studies. For example, Horn et al. [15] looked at patterns of RSV infection in the United States and found that adults from racialized communities or with less access to material resources are more likely to be hospitalized or die from an RSV infection. Other studies corroborated these findings for RSV [16] and COVID-19 [19] within Canada, and for influenza [18] and influenza-like diseases [17, 20] across multiple countries. Other studies specifically focused on Ontario found that COVID-19 incidence was strongly associated with low income or more racially diverse neighbourhoods [27] and that pre-pandemic RSV hospitalization rates were higher in more materially deprived neighbourhoods [28]. Our results complemented these studies by providing a more detailed description of how differences related to material deprivation and diversity varied across the entire age spectrum both pre- and post-pandemic.

Studies have found that the prevalence of respiratory infections is higher in urban compared to rural settings in Canada [22], India [29], China, and Italy [21]. However, differences related to rurality in hospitalizations from respiratory infections varied by geographic location. For example, Zhu et al. showed on a national level that COVID-19 hospitalization rates were greatest in rural areas in the United States [30]; albeit, a study focusing only on the state of South Carolina found no statistically significant differences in COVID-19 hospitalization rates between urban and rural environments [31]. Our results on age-specific hospitalization rates across Ontario, in contrast, showed higher rates among urban residents but only for a subset of age groups. This discrepancy based on geographic location is likely a result of a complex interplay of many effects. For instance, rural residents have more limited access to the healthcare system and, consequently, a lower overall respiratory virus testing rate than urban residents. Rural residents are also less likely to contract infections due to living in less dense populations. There are also myriad socioeconomic mechanisms at play that may further influence the transmission and severity of respiratory viruses, such as occupation, income, housing conditions, and pollution levels.

Our finding of sex-related differences in admission rates was likely influenced by both sex-related biological differences and gender-related differences in health-seeking behaviour. Particularly, several previous studies have shown that males tend to experience a higher level of disease burden than females across many infectious diseases but are less likely to seek healthcare, despite the existence of a universal healthcare system in Ontario [10, 11, 13, 14]. This behaviour could plausibly explain our observations of limited sex differences in hospitalization rates and higher female hospitalization rates in some age groups. Gender-related differences in types of occupations and care-providing roles may also place females at increased risk of hospitalization [32], although this cannot be fully assessed with the dataset studied here.

Our results also suggest that the onset of the COVID-19 pandemic had an impact on the typical seasonal patterns of influenza and RSV, consistent with other studies from Ontario [33, 34], across Canada [35, 36], and internationally [37]. The non-existence of influenza and RSV seasonality in 2020–2021 was a result of the various NPIs in place to combat COVID-19 transmission, as things like masking, social distancing, and air ventilation also reduced the spread of other respiratory pathogens. Larger peak hospitalization rates during the 2022–2023 season may be attributable to the fact that people had less exposure to pathogens during full or partial lockdowns, leaving their immune systems more susceptible to these pathogens once restrictions were lightened [37].

The use of population-based data capturing multiple seasons and all age groups is a key strength of our work, as it allows for an understanding of the burden placed on the Ontario healthcare system by the ‘tripledemic’; however, there are some limitations to consider. Foremost, there may be limitations in our ability to generalize these findings to patterns of viral infection within the broader population, particularly less severe forms of illness. Whereas infections are attributable only to disease spread between individuals, hospitalization rates are influenced by other factors, such as the level of access to healthcare resources. Furthermore, this dataset focuses exclusively on hospitalization rates; it does not allow us to identify overall infection rates or changes in immunity. Weekly hospitalization rates also become too small as we stratify by multiple subgroups to accurately identify interactions between the various subgroups studied. Moreover, while we have provided a comparison of trends pre- and post-pandemic, known changes in the use of multiplex testing following the pandemic will have effects on the observed hospitalization rates [38].

## Conclusion

Our study identified multiple factors such as age, neighbourhood-level material deprivation, neighbourhood-level ethno-racial diversity, rurality, and sex, that impact respiratory virus hospitalization rates. Our study findings can help policymakers to design and implement interventions that can reduce the burden of respiratory viruses.

## Supporting information

10.1017/S0950268826102131.sm001 Mitchell et al. supplementary material Mitchell et al. supplementary material

## Data Availability

The dataset from this study is held securely in coded form at ICES. While legal data-sharing agreements between ICES and data providers (e.g., healthcare organizations and government) prohibit ICES from making the data set publicly available, access may be granted to those who meet prespecified criteria for confidential access, available at www.ices.on.ca/DAS (email: das@ices.on.ca). The full dataset creation plan and underlying analytic code are available from the authors upon request, with the understanding that the computer programs may rely upon coding templates or macros that are unique to ICES and are therefore either inaccessible or may require modification.

## Supplementary material

The supplementary material for this article can be found at http://doi.org/10.1017/S0950268826102131.

## Long descriptions

### Figure 1. Long description

The grid consists of 6 columns (seasons) and 6 rows (age groups). The y-axis represents Weekly Hospital Admission Rates per 10,000 People, with scales varying by row. The x-axis represents the Date from July to July.

* Data Series: Influenza is orange, R S V is magenta, and C O V I D-19 is blue.

* Top Row (Age 0-2): R S V shows the highest peaks, reaching approximately 7.5 in 2022-2023. Influenza peaks are lower, around 1.0 to 2.5. C O V I D-19 remains low until a small peak in 2021-2022.

* Second Row (Age 3-5): Similar patterns to the 0-2 group but with lower overall rates. R S V and Influenza peaks are nearly equal in 2022-2023 at approximately 1.5.

* Middle Rows (Ages 6-12 and 13-18): Influenza typically dominates pre-pandemic seasons. In 2021-2022 and 2022-2023, C O V I D-19 and Influenza show prominent, distinct peaks.

* Bottom Rows (Ages 19-64 and 65+): C O V I D-19 becomes the dominant cause of admissions starting in the 2020-2021 season. For the 65+ group, C O V I D-19 peaks reach between 4.0 and 6.0 in the later seasons, significantly higher than Influenza and R S V which stay below 2.0.

* Seasonal Trends: Pre-2020 seasons show clear winter peaks for Influenza and R S V. The 2020-2021 season shows almost zero Influenza and R S V activity across all ages, replaced by C O V I D-19 waves. In 2022-2023, all three viruses show activity, with an exceptionally early and high R S V peak in the youngest age groups.

Navigate back to Figure 1..

### Figure 2. Long description

A grid of 54 line graphs organized into three age-based sections. The y-axis represents Weekly Hospital Admission Rates per 10,000 People, and the x-axis represents time from July to July across six annual columns from 2017-2018 to 2022-2023.

* Data Series: Orange lines represent high material deprivation and blue lines represent low material deprivation. In almost all panels, the orange line sits vertically higher than the blue line, indicating higher admission rates in deprived areas.

* 0 to 2 years old section: Influenza and R S V show consistent seasonal peaks in winter months. C O V I D 19 panels are flat until 2020-2021, showing a significant peak in 2021-2022.

* 19 to 64 years old section: Admission rates are lower in scale than the youngest group. Influenza peaks are sharpest in 2017-2018 and 2019-2020. C O V I D 19 shows a multi-modal peak structure starting in late 2020.

* 65 plus years old section: Influenza and R S V show regular seasonal curves. C O V I D 19 shows the highest relative admission rates for this age group compared to others, with sustained activity and multiple peaks from 2020 through 2023.

* Trends: R S V activity is nearly zero in the 2020-2021 column across all ages. The gap between high and low deprivation is most pronounced during peak infection periods.

Navigate back to Figure 2..

### Figure 3. Long description

The visualization consists of 54 individual line graphs. The y-axis represents Weekly Hospital Admission Rates per 10,000 People, with scales varying by row. The x-axis represents the Date, spanning July to July for each annual column. Two data series are shown: orange lines for least diverse neighborhoods and blue lines for most diverse neighborhoods.

* 0-2 years old block:

- Influenza: Peaks annually around January. The 2019-2020 season shows the highest peak (approximately 2.5). Rates are near zero in 2020-2021.

- R S V: Shows consistent annual peaks. Orange lines (least diverse) frequently peak higher than blue lines, particularly in 2018-2019 and 2021-2022.

- C O V I D-19: Flat until 2021-2022, where a significant peak occurs in January, with blue lines higher than orange.

* 19-64 years old block:

- Influenza and R S V: Follow similar seasonal patterns to the younger group but at much lower scales (y-axis max 0.3 and 0.1 respectively).

- C O V I D-19: Shows a major peak in 2020-2021 and 2021-2022. In the 2020-2021 winter, the blue line (most diverse) peaks significantly higher (approx 1.4) than the orange line (approx 0.4).

* 65+ years old block:

- Influenza: Peaks are highest in 2017-2018, with the blue line reaching nearly 2.0.

- R S V: Seasonal peaks are generally under 0.6, with orange and blue lines closely aligned.

- C O V I D-19: This row has the highest y-axis scale (up to 10.0). Large peaks appear in 2020-2021 and 2021-2022. In both instances, the blue line for most diverse neighborhoods shows substantially higher admission rates than the orange line.

Navigate back to Figure 3..

### Figure 4. Long description

The graph consists of 54 individual panels. The y-axis represents Weekly Hospital Admission Rates per 10,000 People, and the x-axis represents the Date from July to July. Orange lines and points represent rural residents, while blue lines and points represent urban residents.

* Top Block (0 to 2 years old): Influenza and R S V show consistent seasonal peaks in winter months across all years, with a notable absence of R S V in 2020 dash 2021. C O V I D dash 19 panels are flat until late 2020, showing a major peak in early 2022 where urban rates significantly exceed rural rates.

* Middle Block (19 to 64 years old): Influenza and R S V show much lower admission rates compared to the youngest group. C O V I D dash 19 shows a distinct pattern starting in 2020, with urban peaks in early 2021 and early 2022 reaching approximately 1.0 per 10,000, while rural rates remain lower.

* Bottom Block (65 plus years old): This group shows the highest admission rates for Influenza and C O V I D dash 19. Influenza peaks are highest in 2017 dash 2018 and 2022 dash 2023, with urban rates consistently higher than rural. C O V I D dash 19 shows multiple high-intensity peaks from 2020 onwards, with urban rates peaking near 6.0 in early 2022.

Across most panels, urban (blue) admission rates tend to peak higher and earlier than rural (orange) rates, particularly for Influenza and C O V I D dash 19. Shaded bands around the lines indicate 95 percent confidence intervals.

Navigate back to Figure 4..

### Figure 5. Long description

The graph displays weekly hospital admission rates per 10,000 people. The x-axis represents the date from July to July for each annual column. The y-axis scales vary by row and age group.

* 0-2 years old block: Influenza and R S V show consistent seasonal peaks in winter months across most years, with a notable absence of cases in 2020-2021. C O V I D-19 admissions emerge in 2021-2022 with a sharp peak followed by smaller waves. Male rates (blue) are generally higher than female rates (orange) in this age group.

* 19-64 years old block: Admission rates are significantly lower than other age groups. Influenza and R S V follow seasonal patterns except for 2020-2021. C O V I D-19 shows a large peak in late 2020 and early 2021, with subsequent waves in 2021-2022. Sex differences are minimal.

* 65+ years old block: This group shows the highest admission rates for C O V I D-19, peaking near 7.5 per 10,000 in early 2022. Influenza peaks are highest in 2017-2018. R S V shows steady seasonal peaks around 0.4 to 0.6. Male rates frequently exceed female rates during peak periods.

Across all panels, the 2020-2021 period shows near-zero admissions for Influenza and R S V, coinciding with the rise of C O V I D-19. The 2022-2023 season shows a return to high Influenza and R S V peaks.

Navigate back to Figure 5..
