# Supplementary material for: Social disparities and the COVID-19, influenza, and RSV ‘tripledemic’ in Ontario, Canada: A population-based study
Source: Epidemiol Infect. 2026 Sep 24;154:e117. doi: 10.1017/S0950268826102131 (PMC13749190; doi:10.1017/S0950268826102131)
Supplement: Supplementary file 1 [file S0950268826102131sup001.docx]

Supplementary Material to: Social disparities and the COVID-19, influenza, and RSV “tripledemic” in Ontario, Canada: a population-based study

Figure S1: Weekly age-specific hospital admission rates for high (orange) and low (blue) neighbourhood-level material deprivation. Points represent observed data, while lines and shaded bands represent local regression curves and associated 95% confidence intervals. Note: vertical axis scales differ between rows.

Figure S2: Weekly age-specific hospital admission rates for least (orange) and most (blue) ethno-racially diverse neighbourhoods. Points represent observed data, while lines and shaded bands represent local regression curves and associated 95% confidence intervals. Note: vertical axis scales differ between rows.

Figure S3: Weekly age-specific hospital admission rates for rural (orange) and urban (blue) residents. Points represent observed data, while lines and shaded bands represent local regression curves and associated 95% confidence intervals. Note: vertical axis scales differ between rows.

Figure S4: Weekly age-specific hospital admission rates for female (orange) and male (blue) sex. Points represent observed data, while lines and shaded bands represent local regression curves and associated 95% confidence intervals. Note: vertical axis scales differ between rows.
